# Biases in national and continental flood risk assessments by ignoring spatial dependence

**DOI:** 10.1038/s41598-020-76523-2

**Published:** 2020-11-09

**Authors:** Viet Dung Nguyen, Ayse Duha Metin, Lorenzo Alfieri, Sergiy Vorogushyn, Bruno Merz

**Affiliations:** 1 GFZ German Research Centre for Geosciences, Section Hydrology, 14473 Potsdam, Germany; 2 Institute of Environmental Science and Geography, University of Potsdam, 14476 Potsdam, Germany; 3 European Commission, Joint Research Centre, 21027 Ispra, Italy; 4 CIMA Research Foundation, 17100 Savona, Italy

**Keywords:** Natural hazards, Environmental impact, Hydrology

## Abstract

Recently, flood risk assessments have been extended to national and continental scales. Most of these assessments assume homogeneous scenarios, i.e. the regional risk estimate is obtained by summing up the local estimates, whereas each local damage value has the same probability of exceedance. This homogeneity assumption ignores the spatial variability in the flood generation processes. Here, we develop a multi-site, extreme value statistical model for 379 catchments across Europe, generate synthetic flood time series which consider the spatial correlation between flood peaks in all catchments, and compute corresponding economic damages. We find that the homogeneity assumption overestimates the 200-year flood damage, a benchmark indicator for the insurance industry, by 139%, 188% and 246% for the United Kingdom (UK), Germany and Europe, respectively. Our study demonstrates the importance of considering the spatial dependence patterns, particularly of extremes, in large-scale risk assessments.

## Introduction

Flooding is a major hazard, with global average annual flood loss estimated to US$ 104 billion^1^. Flood damages have been increasing in the last decades^2^ and are projected to increase further, mainly due to a combination of climate change and socio-economic development (e.g. expansion of urban areas and economic growth in flood-hazard zones)^3,4^. In Europe, observed data suggest that climate change has already significantly altered flood magnitude, timing and extent. Blöschl et al.^5^ demonstrate clear regional patterns of both increase and decrease in observed river flood discharges in the past five decades. Blöschl et al.^6^ additionally finds the changing climate shifts timing of European floods. Furthermore, Kemter et al.^7^ highlight the trends in flood extent, i.e. the area simultaneously experiencing peak flows at multiple gauges. They demonstrate the alignment of trends in magnitude and extent. Disaster risk reduction requires to assess flood risk, defined as the relation between the likelihood of flood events and their potential adverse consequences^8–10^. In the last decade, flood risk assessments have been extended to the national and continental scale (e.g. Refs.^2,11,13,14^). These large-scale assessments have often assumed spatially homogeneous flood scenarios, where each area within the large-scale region is subject to an event with the same exceedance probability or return period^12^. For instance, Ward et al.^11^ and Winsemius et al.^2^ at the global scale and Feyen et al.^13^, Rojas et al.^14^ and Bubeck et al.^15^ at the European scale, and te Linde et al.^16^ at the scale of the Rhine basin estimate flood risk in terms of expected annual damage (EAD) and/or expected annual affected population (EAP) under the assumption of homogeneous return periods. Other studies quantify risk in terms of damage or affected population for specific return period floods. Hirabayashi et al.^17^ provide the number of people exposed to 100-year flood assuming homogeneous scenarios at the global scale. For the USA, Wing et al.^18^ estimate damages and number of people exposed to present and future 50-, 100- and 500-year floods. Hall et al.^19^ and Dumas et al.^20^ quantify economic damage and/or number of people exposed to the 100-year flood apart from EAD for England and Wales and for France, respectively. Furthermore, Winsemius et al.^21^ assess economic damages for the 15- and 30-year floods in Bangladesh.

In contrast to the homogeneity assumption, floods show substantial spatial variability in the associated atmospheric, catchment and river network processes, and as a consequence, the return periods of discharge peaks vary considerably along the river, across the catchment and across larger regions (e.g. Ref.^22^). This interplay of different processes in the generation of floods leads to distinct flood regimes, i.e. flood timing and magnitude, and spatially heterogeneous dependence patterns in flood peaks^23–25^. Therefore, the assumption of homogeneous return periods is an unrealistic representation of the flood behaviour^12,26^. This may not be a problem for smaller areas where flood peaks at different locations may be highly correlated. However, at the national or continental scale, the homogeneity assumption may bias regional risk estimates. Given the recent rapid developments in large-scale floods risk assessments and the widespread use of the homogeneity assumption, it is an urgent question whether this assumption introduces significant biases.

There are very few studies which have discussed the effect of spatial dependence on flood risk estimates. Lamb et al.^26^, Wyncoll and Gouldby^27^ and Metin et al.^12^ compare three spatial dependence assumptions: (1) complete dependence, i.e. spatially homogeneous flood scenarios, (2) modelled dependence, i.e. spatially dependent scenarios, attempting to represent the real-world spatial dependence, and (3) complete independence, i.e. flood magnitudes vary randomly in space. These studies suggest that the often-used complete dependence assumption overestimates flood damages for large return periods and underestimate damages for small return periods, whereas the EAD values are marginally affected by spatial dependence according to Metin et al.^12^. However, these studies are limited in scale, as Lamb et al.^26^ and Wyncoll and Gouldby^27^ investigate small regions in England (up to 15,000 km^2^) and Metin et al.^12^ analyze the Elbe catchment in Germany (around 150,000 km^2^). Further, Alfieri et al.^28^ and Jongman et al.^29^ compare risk estimates for the modelled dependence and complete independence assumptions for several European countries and for Europe, respectively. However, they do not explore the widespread assumption of complete dependence. Regional flood risk estimates may also be affected by tail dependence between flood peaks at different locations. If tail dependence exists, for instance, weak correlation between mean values of the random variables but strong correlation between extremes, it needs to be incorporated in multivariate risk assessments^30^. However, the effects of tail dependence have not been sufficiently investigated for regional flood risk assessments.

Here, we develop a multivariate, copula-based statistical model to generate 10,000 years of spatially dependent time series of AMS (Annual Maximum Streamflow) at 379 stations across Europe (“Methods”). These synthetic time series are transferred into inundation areas and economic damages, using the simulation results of Alfieri et al.^31^. Regional risk curves, relating the damage within a given region to its probability of exceedance or return period, are then derived for the three spatial dependence assumptions, i.e. complete dependence, modelled dependence and complete independence. Risk estimates are given for three regions, Europe, Germany and the UK. The latter two are selected due to the high density of discharge stations in these areas. To investigate the effect of tail dependence, we use three copula models with different degree of tail dependence.

## Results and discussion

### Evaluation of the multivariate dependence model

**Figure 1:**
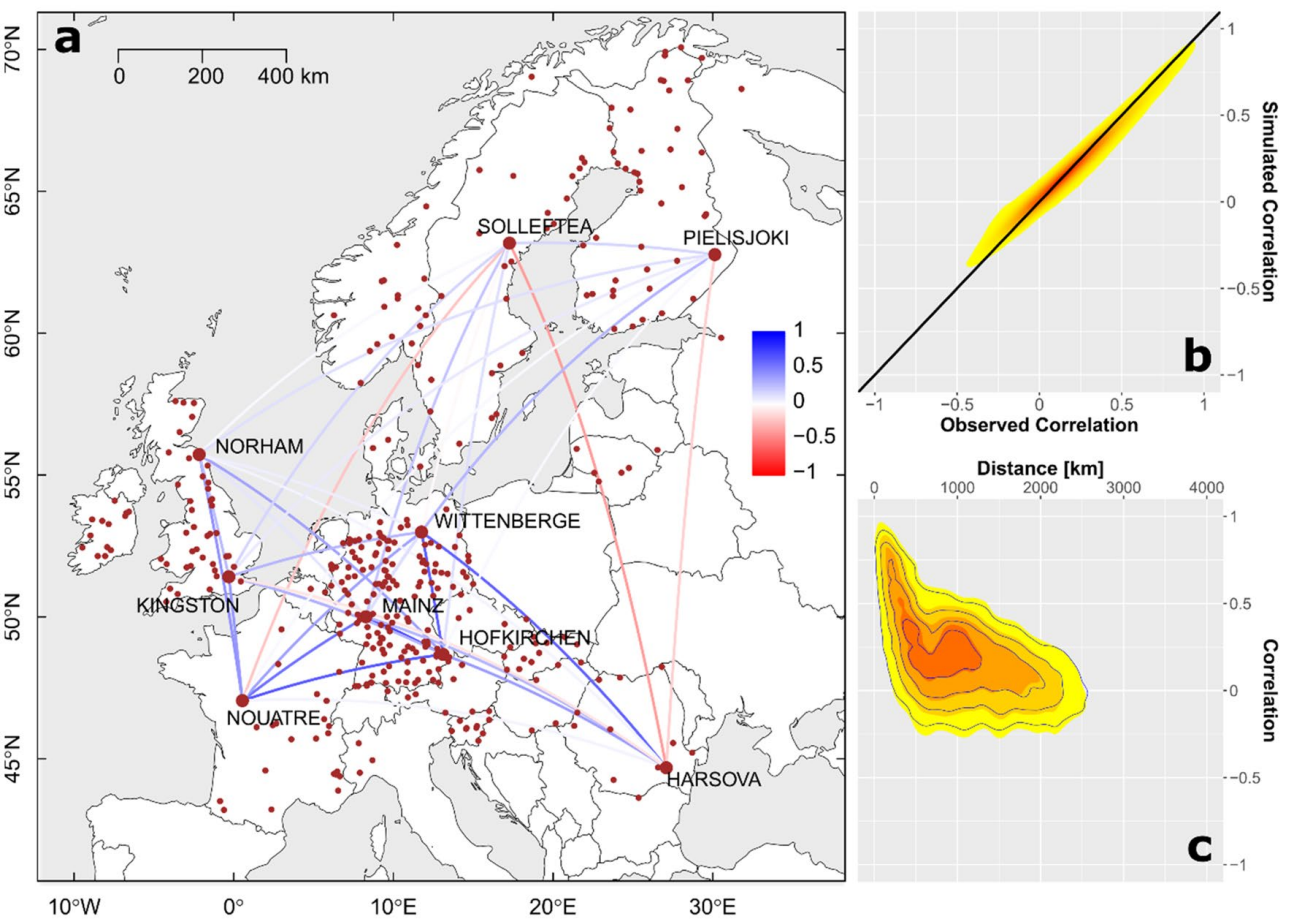
Study area and dependence structure of the AMS data set. (**a)** Locations of 379 gauging stations (red dots) and pairwise correlation (coloured lines) of nine selected stations over Europe. (**b**) Comparison of observed and simulated correlation for all stations. Note the increase of density from yellow to red. (**c**) Correlation versus distance between stations, i.e. correlogram, for observed data (density increases from yellow to red) and simulated data (contour lines). (**b**,**c**) Simulated values are generated by the Student-t copula with *df* = 11.4. All figures created in this paper are based on the free software environment R for statistical computing and graphics (https://www.r-project.org/).

Annual maximum streamflow (AMS) series at 379 gauging stations (Fig. 1a) are extracted from the observational data for the period 1968–1999. These series are used to construct the copula-based multivariate model. The Student-t copula is parameterized using the (379 × 379) correlation matrix and the number of degrees of freedom *df*. The estimated value (*df* = 11.4) indicates a moderate tail dependence in the AMS dataset. The pairwise correlation between AMS series, quantified by Kendall’s tau, varies between − 0.557 and 0.982 with a rapid decline with distance (Fig. 1c). However, there are pairs of stations which are significantly correlated even though they are up to 2000 km apart. The pairwise correlations are visualized exemplarily for nine selected stations (Fig. 1a). We use the Student-t copula model to generate 10,000 years of synthetic AMS series. The agreement between simulated and observed correlation is very good (Fig. 1b,c).

**Figure 2:**
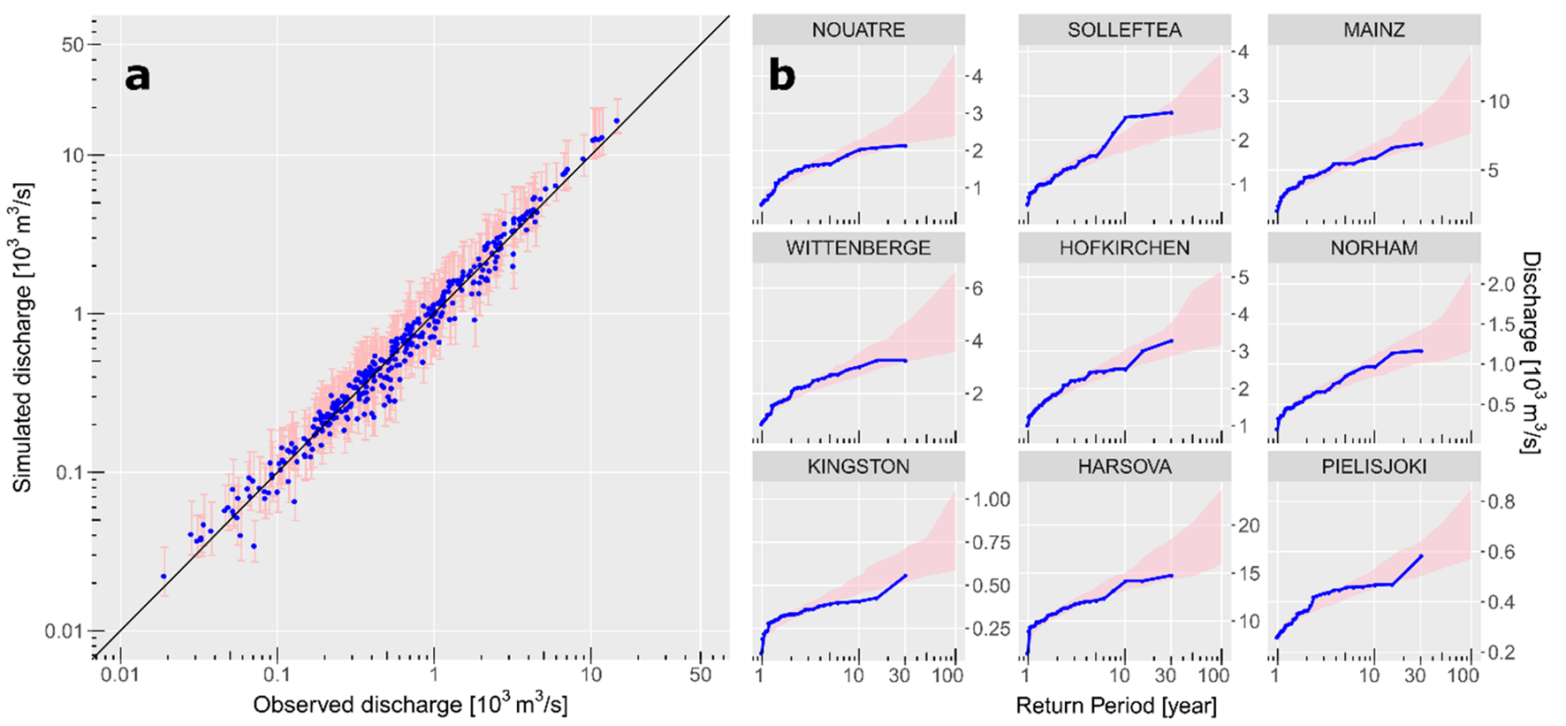
Evaluation of the multivariate dependence model. (**a**) Maximum observed versus simulated peak flow over 31-year period at all stations. Blue dots represent the median of the pink 95% confidence range corresponding to 322 model realizations of 31 years length. Black line represents the identity (1:1) line. (**b**) Flood frequency for nine selected stations (see location in Fig. 1): observations (blue curves) and 95% confidence range (shaded ribbons) corresponding to 100 model realizations of 100 years length.

We fit the Gumbel and the GEV (Generalized Extreme Value) distribution to the observed AMS series at the 379 locations (“Methods”). Two goodness-of-fit tests, Anderson–Darling and Cramer–von Mises, indicate very good fits to the observed AMS series (Supplementary Fig. 1). The multivariate dependence model, i.e. the combination of copulas and marginal distributions, shows good agreement with observations. Figure 2a shows a plausible range of the maximum simulated peak flows over 31-year period at most gauging stations as 87% of confidence range bars cross and the rest deviates slightly from the identity line. Also, the flood frequency curves derived from observed and synthetic discharge correspond well, with the observed flood frequency curves mostly located within the 95% confidence bounds of simulated curves (Fig. 2b).

### Risk estimates for the three dependence assumptions

**Figure 3:**
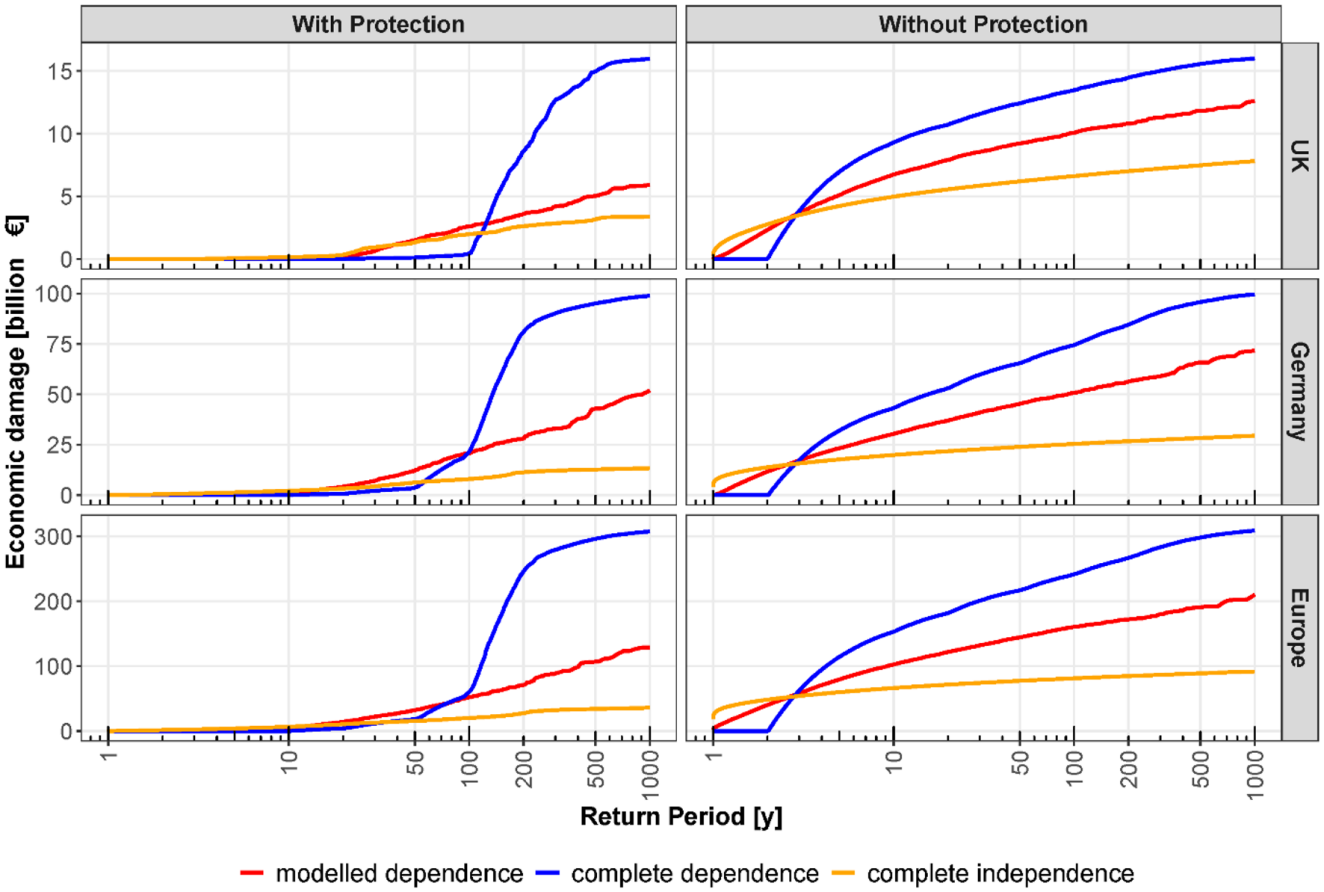
Regional flood risk curves. Flood damages and their corresponding return periods under the assumptions of complete dependence, modelled dependence and complete independence for the UK, Germany and Europe for the scenario with flood protection and without flood protection.

The regional risk curves, i.e. the relation between aggregated flood damages and return periods for the considered regions, are strongly affected by the dependence assumption (Fig. 3). The complete dependence assumption overestimates regional flood risk for large return periods but underestimates risk for small to medium return periods. The shift from underestimation to overestimation, in the following termed the intersection point, occurs roughly around the flood protection levels, i.e. between return periods of 80–120 years for the three regions. The misestimation of risk is explained by the assumption of homogeneity. The complete dependence assumption assigns the same return period discharge peaks to all gauges and to corresponding damages in the adjacent areas. If this return period is smaller than the flood protection level for all (or most of the) areas, the aggregated damage for the region is zero (or small). If it is higher than the protection level, on the other hand, it causes damages in all areas as the protection is overtopped throughout the region. In reality, represented by the modelled dependence assumption, the spatial variability of flood peaks causes damages at some locations even when the regional return period of this event, i.e. the return period of the total aggregated damage, is clearly below the protection level (Supplementary Fig. 2). Hence, the spatial variability leads to a smoothly increasing regional risk curve, compared to the rather threshold-like curve for the complete dependence assumption. The bias by the complete dependence assumption is substantial (Fig. 3). For the 200-year return period, damage is overestimated by 139%, 188% and 246% for the UK, Germany and Europe, respectively. The 50-year damage is underestimated by 93%, 69% and 42%, respectively. The intersection points between the complete independence and the modelled dependence curves have the return period of 38, 15 and 12 years for three regions respectively. The risk curve of the complete independence behaves differently as it shifts from overestimation to underestimation of flood damage at the intersection point compared to modelled dependence moving from low to high return period level. The 200-year flood damage is underestimated by 27%, 60% and 61%, respectively, for the three regions. The regional 50-year damage is still underestimated by 12%, 48% and 52%. However, the 10-year damage is found to be overestimated by 75%, 69% and 14% respectively.

Alfieri et al.^31^ estimate the economic damage for the 100-year flood event as €1.5 billion for the UK, €15 billion for Germany and €120 billion for Europe. Our estimates are somewhat higher at the national scale (€2.6 billion for the UK, €20 billion for Germany), but much lower at the continental scale (€52 billion for Europe). The grid-based simulation model of Alfieri et al.^31^ considers entire Europe, whereas our estimate is limited to the catchments associated with the 379 gauges. Since many areas in Europe are not covered by observational data in the Global Runoff Data Centre (GRDC)^32^, our regional risk estimates consider only part of the entire area for the UK, Germany and Europe, respectively. For the UK and Germany, where we have a high density of stations, our estimates are much closer to the results of Alfieri et al.^31^.

The main influence on the intersection point, i.e. where underestimation turns into overestimation for the complete dependence assumption, is the flood protection level (Fig. 3). For a scenario without protection, the intersection point corresponds to a return period of 3 years. The damage model assumes that there is no damage for discharge peaks below the 2-year flood, which is a good proxy for bankfull conditions^33^. Hence, the risk curves for the complete dependence assumption show damages only for events larger than 2 years. In contrast, the modelled and complete independence assumptions estimate damage also for the 2-year return period, as the spatial variability causes some locations to have peaks higher than the 2-year flood.

### Effects of tail dependence on regional risk estimates

**Figure 4:**
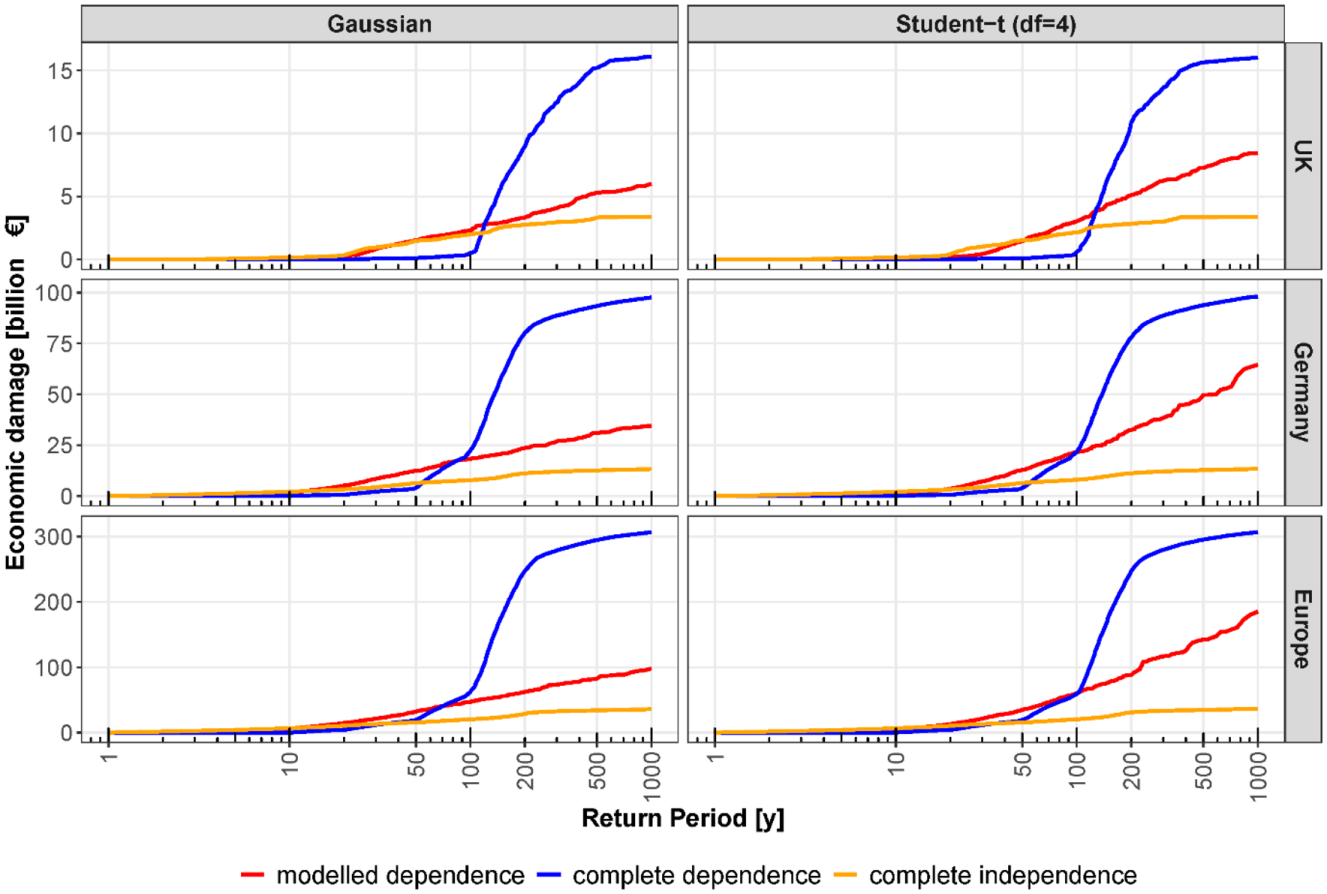
Influence of tail dependence on regional risk curves. Flood damages and their corresponding return periods for the UK, Germany and Europe for the three dependence assumptions. The Gaussian copula does not include tail dependence, while the Student-t copula with *df* = 4 represents rather strong tail dependence.

To understand how the tail dependence affects the regional risk estimates and the biases of the different dependence assumptions, we fit two additional copula models to the AMS data: The Gaussian copula, which does not include tail dependence, and the Student-t copula with *df* = 4. This value is chosen to represent strong tail dependence. A stronger tail dependence leads to higher damage estimates for large return periods, moving the regional risk curve of the modelled dependence assumption closer to the complete dependence assumption (Fig. 4). For the 200-year regional damage, for instance, the overestimation of 139%, 188% and 246% for the UK, Germany and Europe is reduced to 113%, 140% and 180%, respectively, for the scenario with strong tail dependence and increases to 171%, 240% and 298% when removing the tail dependence by assuming the Gaussian copula.

## Conclusions

The study highlights a potential misestimation of flood risk at national and continental scales. We find that the widespread homogeneity assumption overestimates the regional 200-year damage, which is a benchmark indicator for the insurance industry, by 157%, 167% and 233% for the UK, Germany and Europe, respectively. For small return periods, it underestimates flood risk. The intersection point, where underestimation turns into overestimation, depends on the threshold beyond which damages steeply increase, i.e. on the flood protection level. We further show that tail dependence can substantially influence regional risk estimates. The numbers suggest that the misestimation increases with increasing spatial scale. Hence, our study demonstrates the importance of including the spatial dependence of flood peaks and particularly of tail dependence in national and continental risk assessments.

## Methods

### Multivariate dependence model

We adopt a copula-based multivariate model to represent the spatial dependence structure of annual maximum streamflow (AMS) of daily discharge at multiple locations over Europe. The copula approach is based on Sklar’s theorem^34^, which sets up a link between a joint distribution and its marginal distribution functions. One key advantage of the approach is that it can separate the dependence structure from the marginal distributions^35,36^. Among the different classes of copulas, elliptical copulas offer convenience in model construction and computation of high dimensional problems and have close relation to the classical multivariate method^37,38^. We apply the Gaussian and Student-t copulas which are the most widely used elliptical copulas. Both are symmetrical copulas. The Gaussian copula is completely determined by the correlation matrix as its mere parameter which is relatively simple to estimate. However, it lacks tail dependence which measures the co-movement in the tail parts of the distribution. To overcome this shortcoming, the Student-t copula can be seen as an extension of the Gaussian copula as it retains the use of correlation structure and introduces an additional parameter, the degree of freedom (*df*) which supports the co-movement in extreme behaviour. The Student-t copula therefore has tail dependence. The tail dependence of the Student-t copula gets weaker with a higher *df*. In the limiting case where *df* approaches infinity, Student-t copula becomes Gaussian copula.

In this study, the correlation matrix of the Gaussian copula is estimated by the method of moments based on Kendall’s tau. For the Student-t copula, we use the method of Mashal and Zeevi^39^, which combines the method of moments based on Kendall’s tau for estimating the correlation matrix and the maximum pseudo-likelihood-like estimation for determining the number of degrees of freedom. Particularly for a large number of variables, as in our case, the correlation matrix can be estimated incorrectly (not positive definite) due to the truncation error and/or missing data. Therefore, we correct the correlation matrix by the algorithm nearPD (nearest positive definite matrix^40^) available in the package Matrix of the R programming language.

For marginal distributions, we fit the Gumbel distribution to 379 AMS time series using the maximum likelihood method^41^ then test the goodness-of-fit using Anderson–Darling (AD) test^42^ and Cramer–von Mises (CvM) test^43^. Gumbel distribution is preferred due to its simple structure. At 372 stations the fitting passes the tests. We then fit the Generalized Extreme Value (GEV) distribution to data at the remaining 7 stations. Supplementary Fig. 1 shows that all testing p-values are larger than the significance level of 0.05 (with median p-value of 0.81 for the CvM test and 0.84 for the AD test) indicating good fitting at all stations.

### Discharge data and simulation of AMS at multiple locations

Based on daily discharge data with at least 50 years of continuous data from GRDC^32^, we derive AMS time series for a common, 31-year time period (1968–1999). We consider 379 gauging stations in 21 European countries (Fig. 1a). The station geo-location is matched to the 5-km gridded river network of the European Flood Awareness System (EFAS, see Ref.^44^), using criteria based on proximity, naming, and a maximum error between modelled and official upstream area of 20%. In addition, stations with upstream area smaller than 500 km^2^ are excluded, so that discharge peaks can be linked to the corresponding inundated area at 100 m resolution for different return periods^31,45^. The area threshold of 500 km^2^ is the minimum upstream area simulated in the considered JRC European inundation maps, which we use for damage estimation. The copula-based model is used to generate 10,000 years (100 realizations × 100 years) of AMS at the 379 stations.

### Damage calculation from AMS series

The 10,000-year synthetic AMS are used to calculate flood damage. In a first step, AMS values are associated with the maps of flood depth and extent at 100 m resolution. For this, the relation between discharge peaks and return periods are estimated by the Gumbel distribution using the L-moments approach for parameter estimation^46^. Only discharge peaks exceeding the 2-year return period, which is a good proxy for bankfull discharge^33^, are taken into account for damage estimation. The linkage between discharge peaks and inundation depths is obtained from previous 2D hydraulic simulations with the LISFLOOD-FP model^31^. The maximum water depths for selected flood return periods are computed using synthetic flood hydrographs consistent with the flow duration curve at each 5 km river section along the European river network. Flood depth and flood extent at 100 m resolution are estimated on the basis of the CCM Digital Elevation Model^47^. Roughness coefficients for the LISFLOOD-FP model are linked to the 100 m resolution land use map of Europe Batista e Silva et al.^48^.

In a second step, direct economic damage for all economic sectors (i.e. residential, commerce, industry, transport, infrastructure, agriculture) is estimated using the flood maps and country-specific depth-damage functions, given by Huizinga^49^ for different land use classes. Regional differences in asset values for a given land use class are considered by rescaling the depth-damage functions with the GDP (Gross Domestic Product) Purchasing Power Standards of 2007. The damage for selected return periods (T = 10, 20, 50, 100, 200, 500 years) is assessed at 100 m resolution and then aggregated to 5 km resolution through the method of Areas of Influence (AoI), described in Alfieri et al.^31^. Flood damage is calculated upstream of each river station for two scenarios, i.e. with and without flood protection. For the scenarios with flood protection, the damage is set to zero if the return period of the discharge peak is smaller than the flood protection level for the corresponding river section. For details on the economic impact assessment see Refs.^28,31^. Finally, we calculate economic damages on the European scale over 10,000 years by interpolating and extrapolating for AMS values with return periods larger than 500 years. Our damage estimates do not consider the complete European area (1) as the flood maps cover only river catchments larger than 500 km^2^, (2) as the impact model cannot be run due to data limitations in some parts of Europe, e.g. in Iceland, Switzerland, Russia and a few countries in the Balkans, and (3) as significant parts of Europe are not covered by observational gauge data in GRDC database. Hence, our damage estimates cover part of the three regions the UK, Germany and Europe which are selected for the presentation of the results.

### Flood risk assessment for different spatial dependence assumptions

We compute direct flood damages and risk curves for three regions (the UK, Germany, Europe) and for three spatial dependence assumptions: modelled dependence, complete dependence, and complete independence. The modelled dependence assumption mimics the real-world spatial variability of flood peaks and damages across Europe. For each year of the synthetic time series (10,000 years) generated with the copula-based, spatial dependence model, the damage values within the considered region are aggregated. The risk curve of the region is then derived from the empirical cumulative distribution function of these aggregated damage values. Hence, the damage values are directly used to calculate exceedance probabilities, or return periods, shown as regional risk curves in Figs. 3 and 4. This step, i.e. the derivation of the risk curves, is performed in the same way for the other two scenarios. However, for the complete dependence and complete independence scenarios, the simulated spatial correlation is destroyed before aggregating the catchment damage values to regional values. For the complete dependence scenario, it is assumed that in a given year each river station experiences a flood with the same return period at the respective discharge gauge. To this end, the damage values at each gauge are ranked according to their magnitude, and then aggregated for each year. The complete independence scenario assumes that there is no spatial correlation between the flood magnitudes at different stations. Hence, the damages for the 10,000-year time series at each river station are independently shuffled before aggregation. Because this regional estimate depends on the shuffling, we repeat this procedure 100 times. To represent the risk curve, we use the median of the 100 realizations.

Regional flood risk curves are calculated for three dependence models (Gaussian and Student-t copulas, the latter with two variants regarding the number of degrees of freedom), for three regions (the UK, Germany and Europe) and for two protection scenarios (with and without flood protection). The tail dependence affects only the regional risk curves of the modelled dependence assumption, but has no influence on the risk curves for the complete dependence and complete independence assumptions. For the special case, where one is only interested in the EAD, the spatial dependence can be ignored^12^. The scenario without flood protection gives an estimation of the maximum damage under failure of all flood protection measures. Although this scenario grossly overestimates the risk, it indicates the exposed assets protected by flood defences. The scenario with flood protection provides the damage when the flood defences work up to their design levels. Flood protection levels are taken from Jongman et al.^29^.

## Supplementary information

Supplementary Figures.

## Data Availability

The GRDC discharge dataset was obtained from the Global Runoff Data Centre, 56068 Koblenz, Germany (https://www.bafg.de/GRDC/EN, last access: October 2017) and was recently made available for online download via https://portal.grdc.bafg.de. Flood hazard maps for the European Union can be downloaded from https://data.jrc.ec.europa.eu/collection/floods. Flood protection levels are taken from Jongman et al.^29^.
