# Supplementary material for: Biases in national and continental flood risk assessments by ignoring spatial dependence
Source: Sci Rep. 2020 Nov 9;10:19387. doi: 10.1038/s41598-020-76523-2 (PMC7653947; doi:10.1038/s41598-020-76523-2)
Supplement: Supplementary file 1 — Supplementary Figures. [file 41598_2020_76523_MOESM1_ESM.docx]

**Biases in national and continental flood risk assessments by ignoring spatial dependence**

Nguyen Viet Dung^1^, Ayse Duha Metin^1,2^, Lorenzo Alfieri^3,4^, Sergiy Vorogushyn^1^, Bruno Merz^1,2^

^1^GFZ German Research Centre for Geosciences, Section Hydrology, 14473 Potsdam, Germany

2Institute of Environmental Science and Geography, University of Potsdam, 14476 Potsdam, Germany

^3^European Commission—Joint Research Centre, 21027 Ispra, Italy

^4^CIMA Research Foundation, 17100 Savona, Italy

Correspondence and requests for materials should be addressed to N.V.D (email: dung@gfz-potsdam.de)

# Supplementary information


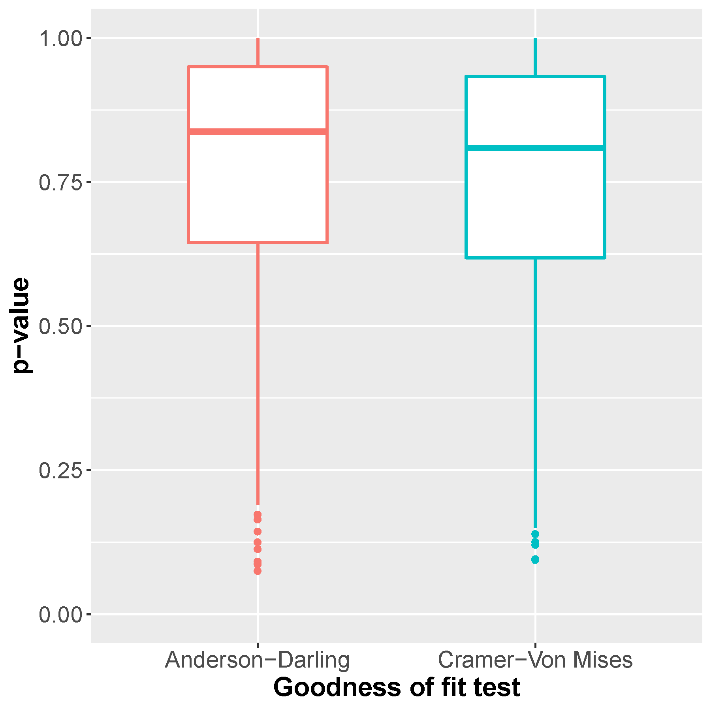


**Suppermentary Figure 1**: p-values of goodness-of-fit tests for fitting the distribution (Gumbel/GEV) to the AMS data. The null hypothesis H_0_ is that the data follow the distribution. The alternative hypothesis H_a_ is that the data do not follow the distribution. The significance level is set at 0.05.


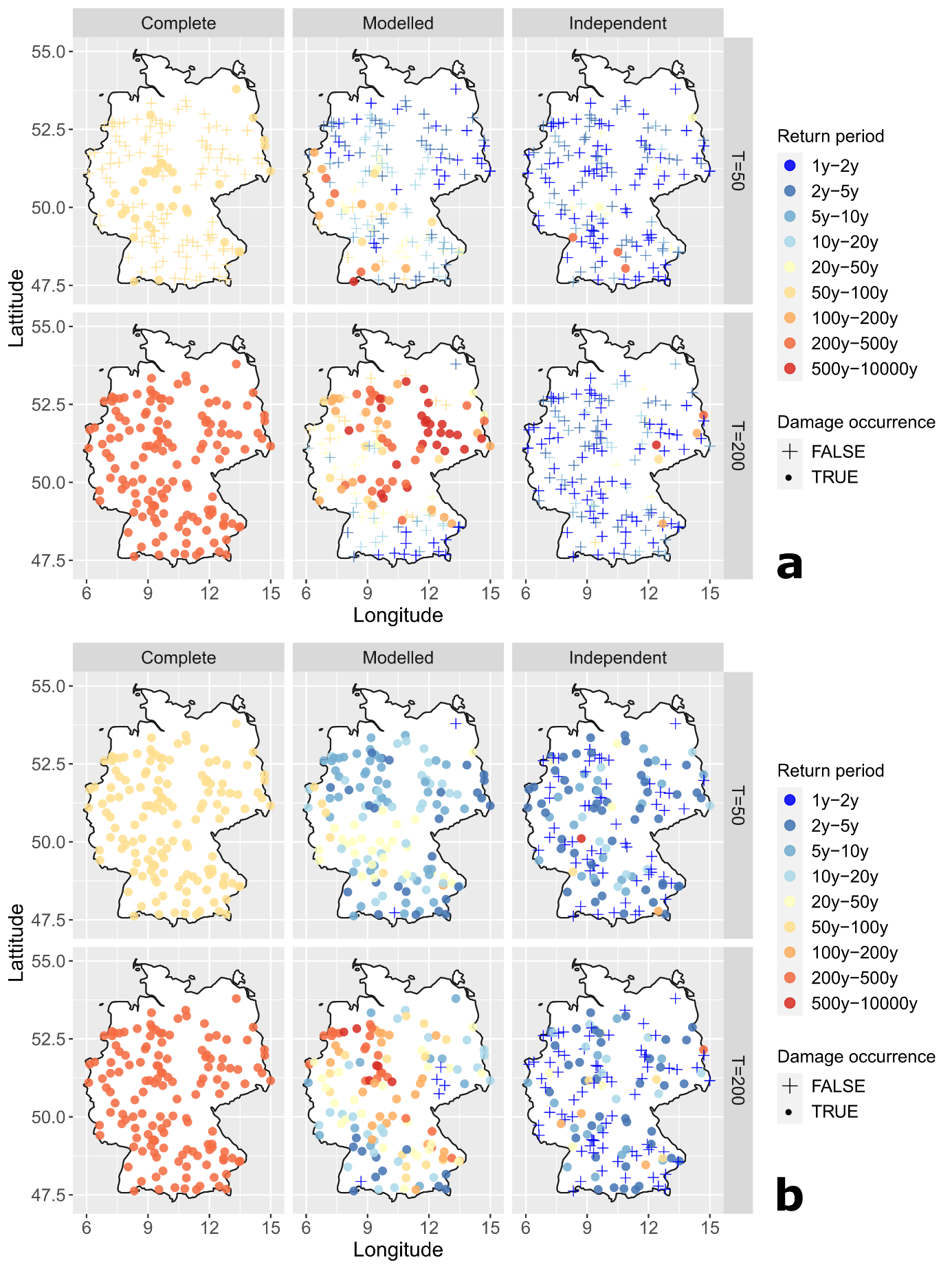


**Supplementary Figure 2**: Spatial distribution of the return period of loss for Germany under the three assumptions: complete dependence, modelled dependence and complete independence. Two return period levels T = 50 years (small) and T = 200 years (high) are selected for illustration. **a** Scenario with flood protection. **b** Scenario without flood protection.
